# Supplementary material for: Pathological Changes in Extracellular Matrix Composition Orchestrate the Fibrotic Feedback Loop Through Macrophage Activation in Dupuytren’s Contracture
Source: Int J Mol Sci. 2025 Mar 28;26(7):3146. doi: 10.3390/ijms26073146 (PMC11988646; doi:10.3390/ijms26073146)
Supplement: Supplementary file 1 [file ijms-26-03146-s001.zip › ijms-3528621-supplementary/Supplementary_Information_IJMS.pdf]

Supplementary Information

Pathological changes in extracellular matrix composition orchestrate the fibrotic feedback loop through macrophage activation in Dupuytren's contracture

Elizabeth Heinmäe[1,2], Kristina Mäemets-Allas[1], Katre Maasalu[3,4], Darja Vastšjonok[1] and Mariliis Klaas[1#]

[1]Institute of Molecular and Cell Biology, University of Tartu, Riia 23b, 51010, Tartu, Estonia

[2]Department of Cell and Molecular Biology, Karolinska Institute, Stockholm, Sweden

[3]Department of Traumatology and Orthopedics, Institute of Clinical Medicine, University of Tartu, 51010 Tartu, Estonia

[4]Clinic of Traumatology and Orthopedics, Tartu University Hospital, 51010 Tartu, Estonia

#To whom correspondence should be addressed: Mariliis Klaas, Institute of Molecular and Cell Biology, University of Tartu, Riia 23b, 51010, Tartu, Estonia; phone: +372 737 5883; email: mariliis.klaas@ut.ee. ORCID 0000-0003-2457-5928.

Supplementary Methods

Proteomics analysis

Precipitation of 15 µg of proteins was carried out using trichloroacetc acid (TCA) and Na-deoxycholate (DOC). Protein pellets were suspended in 30 µL of 7 M urea, 2 M thiourea, 100 mM ammonium bicarbonate (ABC) and 20 mM methylamine solution, followed by disulphide reduction and cysteine alkylation with 5 mM dithiotreitol and 10 mM chloroacetamide for 60 min

at RT. Digestion of proteins with 1:50 (enzyme:protein ratio) Lys-C enzyme was performed for 1 h at 25°C, followed by dilution of the mixture 5 times with 100 mM ABC and further digestion with trypsin overnight at 25C. The next day the digested proteins were injected to an Ultimate 3000 RSLCnano system (Dionex) using a C18 cartridge trap-column in a backflush configuration and an in-house packed 3 µm C18 particles (Dr Maisch) analytical 50 cm × 75 µm emitter-column (New Objective). The same column as above, attached to Dionex Ultimate3000 RSLC nano-LC, was used to separate the peptides. A linear 120 min 8-35% gradient of solvent B (flow rate 250 nl/min) was used to elute the peptides. The peptides were detected with Thermo Fisher Scientific Q Exactive HF mass spectrometer. Each 350-1400 m/z MS scan at a resolution setting 60 0000 was followed by MS/MS analysis of up to 12 most intense peaks. Raw data files were processed using MaxQuant software package (version 2.0.3.0) using default settings. Peptide identification search was performed against the human reference proteome database downloaded in 20.09.2020 from the UniProtKB database.

RT-qPCR

2-5 Dupuytren contracture and Control patient samples were used in growth medium to stimulate macrophages. Total RNA was extracted from macrophages using NucleoSpin RNA mini kit (Macherey-Nagel), according to the manufacturer’s instructions. Reverse-transcription was conducted with a RevertAir First Strand cDNA Synthesis kit (Thermo Scientific) according to manufacturer’s instructions. qPCR analysis was performed using LightCycler® 480 II (Roche). HPRT values were used for normalisation.

Primer sequences were as follows:

HPRT forward primer 5'–CCTGGCGTCGTGATTAGTGATGATGA–3'

HPRT reverse primer 5'–CTTGAGCACACAGAGGGCTACAATG–3'

IL-6 forward primer 5'–GGTGTTGCCTGCTGCCTTCC–3'

IL-6 reverse primer 5'–TGTGTGGGGCGGCTACATCT–3'

TNF-α forward primer 5'–CATCCAACCTTCCCAAACGCCT–3'

TNF-α reverse primer 5'–GGTCTCCAGATTCCAGATGTCAGGG–3'

IL-1β forward primer 5'–CTGGACCTCTGCCCTCTGGATG–3'

IL-1β reverse primer 5'–TGCCTGAAGCCCTTGCTGTAG T–3'

IL-10 forward primer 5'–TGAGAACCAAGACCCAGACATCAAGG–3'

IL-10 reverse primer 5'–AAGGATTCTTCACCTGCTCCAC–3'

TGFβ forward primer 5'–ATTCCTGGCGATACCTCAGCAACC–3'

TGFβ reverse primer 5'–CTCAACCACTGCCGCACAACTC–3'

Supplementary Tables

Supplementary Table S2. Patient tissue samples used in proteomics analysis

Sample	Sex	Age
Control 1	F	48
Control 2	F	71
Control 3	F	75
Control 4	M	75
Control 5	M	61
Control 6	F	88
Control 7	M	64
Control 8	M	53
Control 9	M	72
Control 10	F	69
Control 11	F	82
Control 12	F	73
Control 13	F	72
Control 14	M	74
Control 15	M	74
Control 16	F	60
Control 17	F	57
Control 18	F	44
Control 19	F	44

Control 20	F	86
Dupuytren 1	M	58
Dupuytren 2	M	46
Dupuytren 3	M	56
Dupuytren 4	M	60
Dupuytren 5	M	69
Dupuytren 6	F	74
Dupuytren 7	M	43
Dupuytren 8	F	71
Dupuytren 9	M	74
Dupuytren 10	M	54
Dupuytren 11	M	73
Dupuytren 12	F	67
Dupuytren 13	F	62
Dupuytren 14	M	49
Dupuytren 15	M	55
Dupuytren 16	M	61
Dupuytren 17	M	69
Dupuytren 18	M	69
Dupuytren 19	F	64
Dupuytren 20	M	64

Supplementary Table S3. Patient tissue samples used in immunofluorescence analysis

Sample	Sex	Age
Control 1	M	47
Control 2	F	66
Control 3	F	82
Control 4	F	54
Control 5	M	40
Control 6	M	79
Control 7	F	87
Dupuytren 1	M	48
Dupuytren 2	M	59
Dupuytren 3	M	64
Dupuytren 4	M	74
Dupuytren 5	M	76
Dupuytren 6	M	79
Dupuytren 7	M	75

Supplementary Table S4. Antibodies used in immunofluorescence analysis

**Target**	**Host**	**Dilution**	**Manufacturer; Cat. No.**
ANGPTL2	Rabbit	1:100	Proteintech; 12316-1-AP
CMA1	Rabbit	1:1000	Atlas antibodies; HPA052634
EFEMP1	Rabbit	1:50	Atlas antibodies; HPA070841
FBN1	Rabbit	1:500	Atlas antibodies; HPA021057
αSMA	Mouse	1:50	Leica Biosystems; PA0943
αSMA	Mouse	1:500	Thermo Fisher; 14-9760-82
CD68	Rabbit	1:100	Thermo Fisher; PA532330
CD68	Mouse	1:100	eBioscience; 12-0681
Ki67	Rat	1:200	eBioscience; 14-5698-82
Collagen I	Rabbit	1:300	Abcam; ab34710
MFAP4	Rabbit	1:100	Proteintech; 17661-1-AP
Periostin	Rabbit	1:100	Proteintech; 19899-1-AP
VWA1	Rabbit	1:100	Proteintech; 14322-1-AP
Mouse IgG (Alexa Flour 647)	Donkey	1:1000	Thermo Fisher; A-31571
Mouse IgG (Alexa Flour 488)	Donkey	1:1000	Thermo Fisher; A-21202
Rabbit IgG (Alexa Flour 647)	Donkey	1:1000	Thermo Fisher; A31573

Rabbit IgG (Alexa Flour 488)	Donkey	1:1000	Thermo Fisher; A-11034
Rat IgG (Alexa Flour 594)	Donkey	1:1000	Thermo Fisher; A-21209

Supplementary Figures

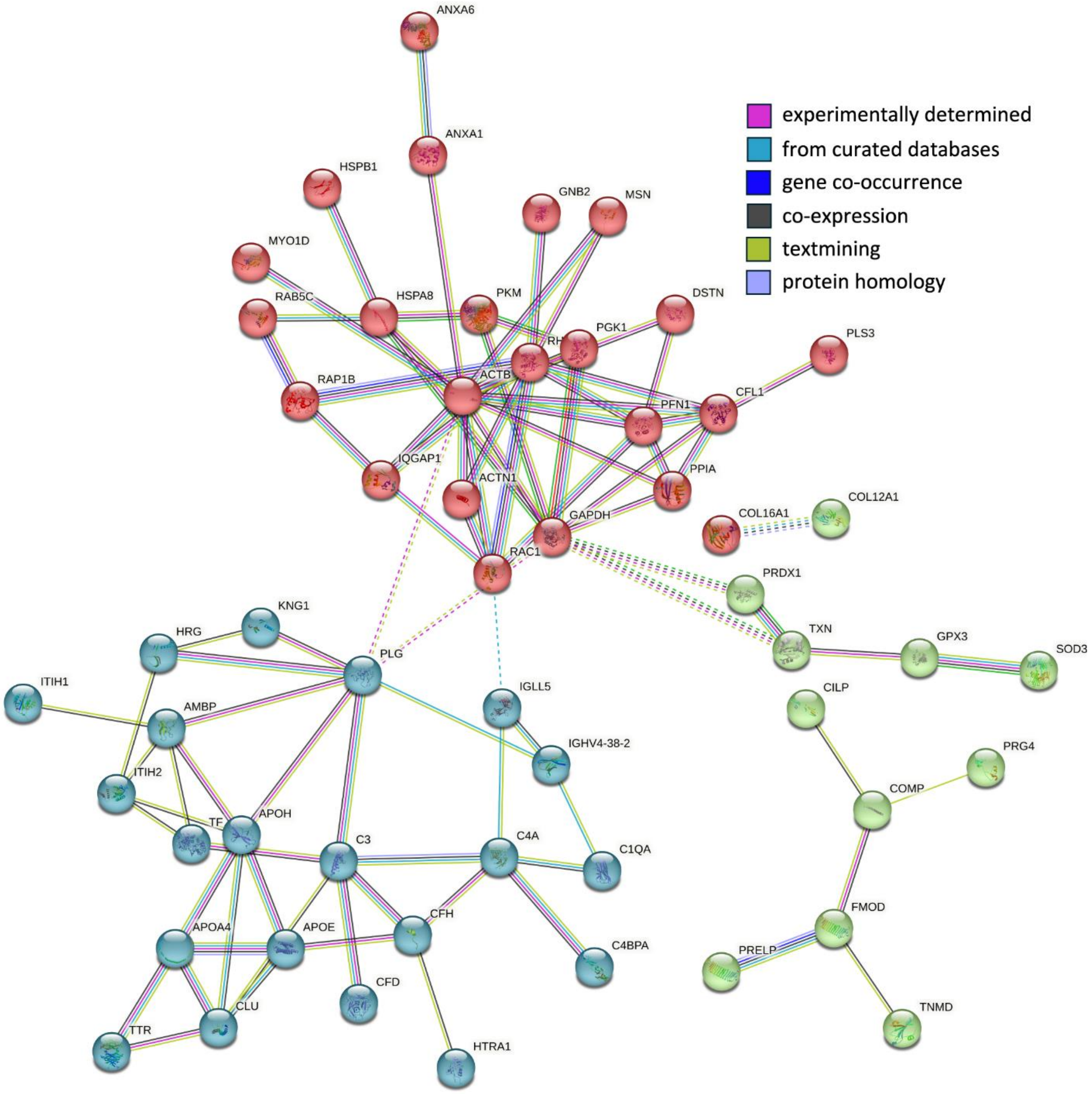

Supplementary Figure S1. Protein-protein interactions between the proteins downregulated in Dupuytren's disease. STRING analysis was conducted with a high confidence threshold 0.7 and the results were clustered using k-means clustering. The interactions between different clusters are visualized by dotted line, while intra-cluster interactions are shown with solid lines. The interactions shown are from curated databases (light blue), experimental data (pink), textmining (green), co-expression (black), gene co-occurrence (dark blue) and protein homology (purple).

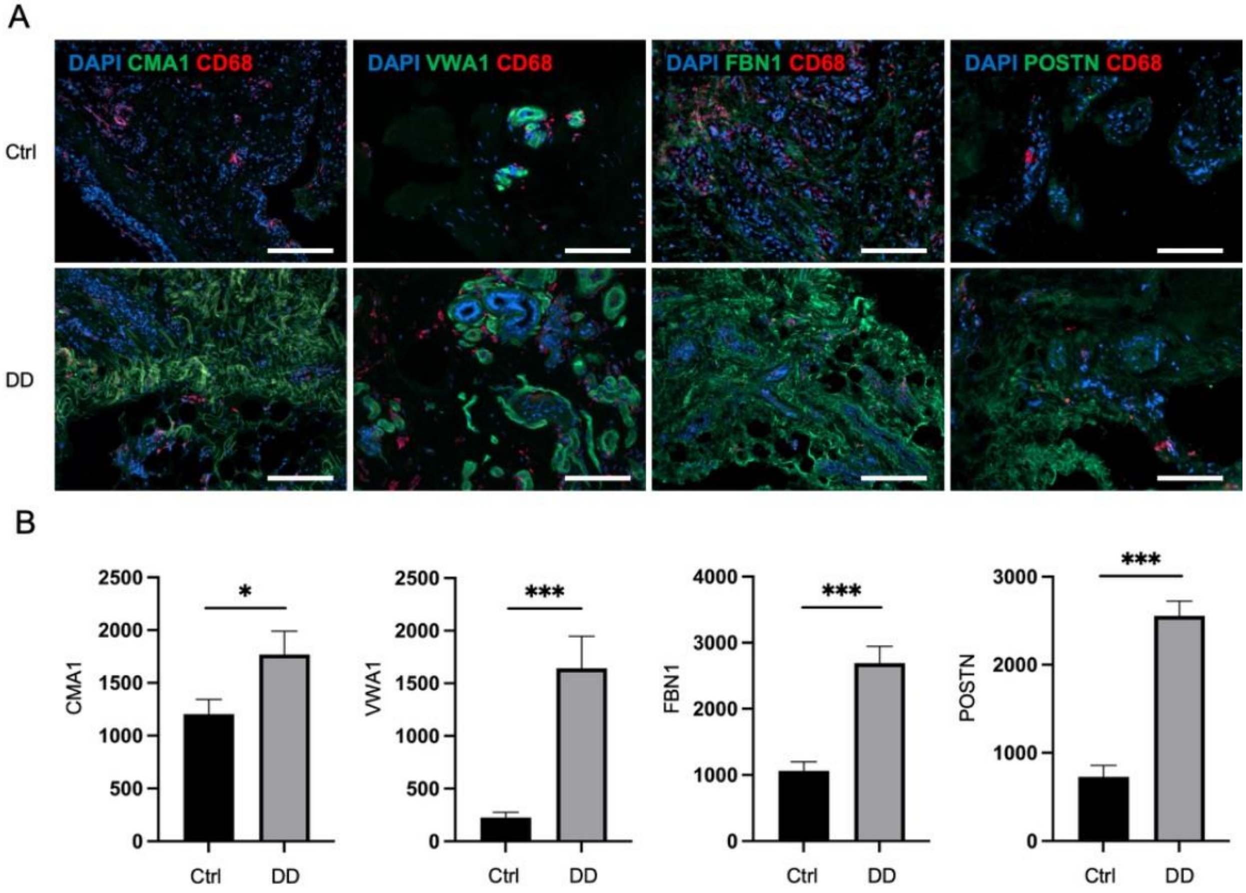

Supplementary Figure S2. Immunofluorescence analysis of DD-associated markers in patients' tissue sections. The representative samples (A) and relative quantification of the fluorescence signal of CMA1, VWA1, FBN1 and POSTN expression by mean integrated density (B) are shown, n = 6-7. The scale bar is 200 µm. *$p < 0.05$; ***$p < 0.001$.

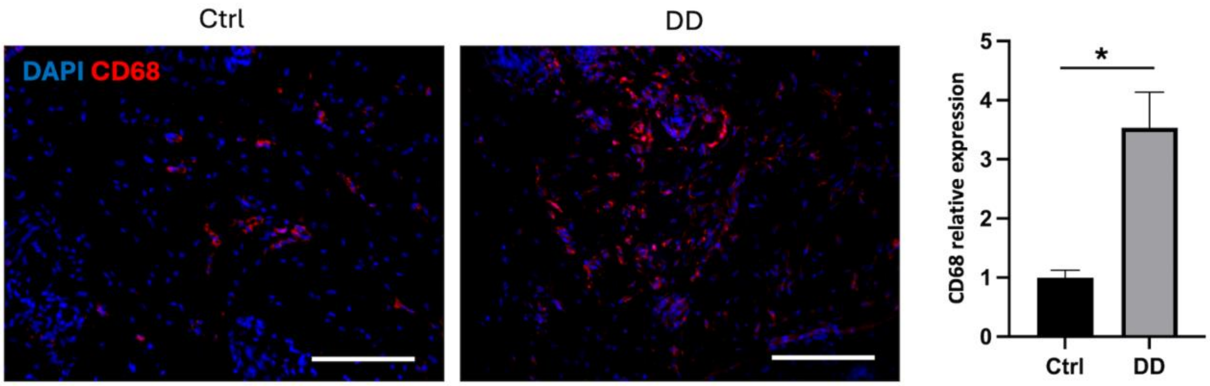

Supplementary Figure S3. Immunofluorescence analysis of the macrophage marker CD68 in DD and control patients' tissue sections. The representative images and relative quantification of the fluorescence signal of CD68 expression are shown, n = 7. The scale bar is 200 µm. *indicates a statistically significant ($p<0.05$) difference.

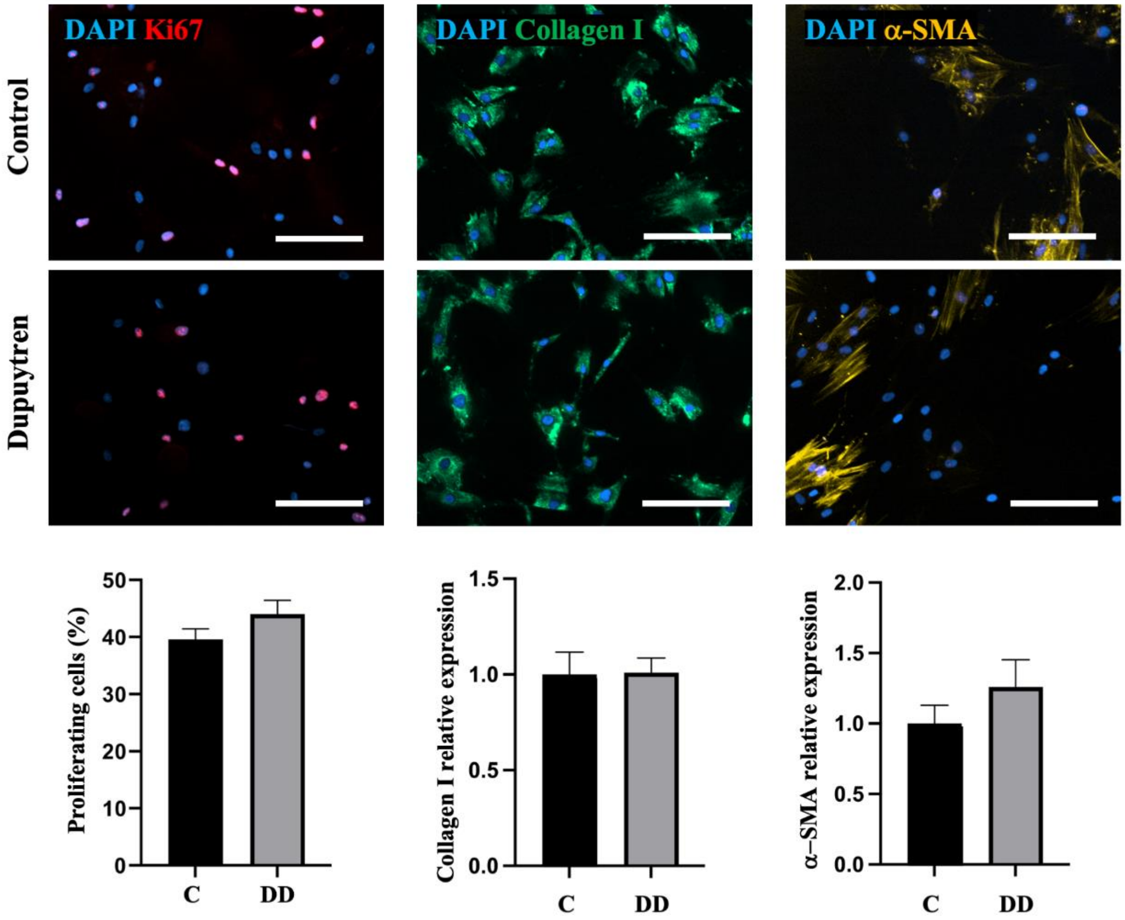

Supplementary Figure S4. DD ECM has no effect on fibroblast proliferation and differentiation into myofibroblasts. Fibroblasts were cultured in cell culture medium containing DD or control palmar fascia ECM for 48h and Ki-67-antigen, type I collagen and α-SMA expression was analyzed using microscopy, n=3. The scale bar is 200 μm.
